# First result of differentiated communication—to smokers and non-smokers—in order to increase the voluntary participation rate in lung screening

**DOI:** 10.1186/1471-2458-13-914

**Published:** 2013-10-02

**Authors:** Mariann Moizs, Gábor Bajzik, Zsuzsanna Lelovics, Marianna Rakvács, János Strausz, Imre Repa

**Affiliations:** 1 “Moritz Kaposi” General Hospital, Kaposvár, Hungary; 2 Institute of Diagnostic Imaging and Radiation Oncology, Health Center, Kaposvár University, Kaposvár, Hungary

**Keywords:** Lung screening, Lung cancer, Smoking, Communication strategy

## Abstract

**Background:**

Lung cancer is the most common fatal malignacy and also the primary cause of cancer mortality. Participation in lung screening is an important step in diagnosing patient in early stage and it can promise better outcomes. The aim of this preliminary study was to determinate the differences in the participation rate of smokers and non-smokers in lung cancer screening and to determine the communication strategies to increase the participation rate.

**Methods:**

In the given period of time (from May to August 2012) out of 1426 people who participated in the lung screening program 1,060 adult volunteers (331 males and 729 females, average age 54.0±9.3 years), completed fully and anonymously author’s questionnaire that contained 28 questions. 25.7% of the respondents were smokers (n=272), 64.6% have never smoked, while 9.7% were former smokers.

**Results:**

Mostly former smokers considered lung screening as an effective method for early detection of pulmonary diseases (86.4%). The most important source (41.0%) of information was the general practitioner. The participation rate of non-smokers is higher in lung screening than the ratio of non-smokers in the population. The unclear data suggest that smokers need distinct, concise messages to know why they should regularly undergo lung screening and doctors have a major role in this.

**Conclusions:**

We found that smokers significantly more frequently took part in lung screening annually. It is positive that the participation rate of former smokers is higher than non-smokers, it is just a bit lower than the participation rate of smokers—both in annual and biannual participation. The participation rate of non-smokers is higher in lung screening than the rate of non-smokers in the population.

## Background

Lung cancer is the leading cancer diagnosis worldwide, since 1985 [1]. Lung cancer is the most common cause of cancer mortality worldwide, its leading position strengthened according to trends [2-5]. Approximately 80-90% of the patients are smoking [6]. 75% of the patients have incurable, locally advanced or metastatic cancer when it is diagnosed. The five year survival rate is 60% for stage I lung cancer, it is 5% for stage IV lung cancer. Only 15% of the patients survive more than five years [7]. Lung cancer is the seventh leading cause of deaths among solid tumors in non-smokers. 15% of lung cancer patients have never smoked. In non-smokers lung cancer is more frequent in women and familial clustering is also more typical and the incidence of adenocarcinoma is higher than squamous cell carcinoma [8]. Lung cancer is a public health problem in developed countries; and it has been shown that the worst situation is in Hungary in all countries [9].

While professionals have long discussions about *“to screen or not to screen” it is* worth to pay attention to this problem from a completely different aspect. If we decide to screen, a major challenge for professionals is to reach that the more people take part in the screening from the risk group. It is a major health science and communication task to convince the target groups and as a result to reach a higher participation rate in the screening programs.

The lung screening program was introduced in Hungary in 1960 for screening Tuberculosis (TB), using chest X-ray. Since the occurrence of the disease has decreased in the past decades the meaning of the screening program has also changed. Instead of TB currently one of the main aims of the program is to detect lung cancer.

The program provides opportunity for the people aged over 40 years to take part in the screening annually. All arising expenses are covered by the Hungarian National Health Insurance Fund.

Today, in lung cancer screening more and more professionals vote for low-dose CT screening [10] according to methodological aspects, however a number of parameters do not have uniform criteria: the age of included people varies in different countries [11,12] (in Hungary it is between 40 and 65 years), the interval of screening also varies: one or two [13] year intervals can be found.

In general, the health economic aspects of expenditures for preventive screening, including lung cancer screening, are analyzed to show the health gains of the certain expenditures for the insurance companies. (Authors state that the value of human life cannot be expressed by money or numbers). At the time of the allocation of scarce resources, in many cases a decision must be taken that a curative and preventive care form receives support, while another do not get funding. It is very important that preventive screenings will be more and more efficient from the first step of the process.

From prevention programs any feedback is rarely made, the effectiveness of these programs contains a number of random, unpredictable elements.

In Central and Eastern European countries researchers calculated the cumulative risk of lung cancer in former smokers. According to their results quitting smoking before the age 50 can decrease the risk of lung cancer with 67-83%. These results show that quitting smoking has a very important role in the reduction of lung cancer caused mortality risk [14] and prevention programs have a major role in this issue. The results of international studies indicate that a screening program can also be an effective tool in successful smoking cessation.

In the United States Zafar et al. analyzed the effectiveness of lung cancer screening on smokers and former smokers. In the prospective study low-dose CT was performed. 22% of the participants had previously cancer, 83% had family history of cancer and 52% of these were lung cancer. 47% of the participants were former smokers. 11% of the smokers had quit smoking and 45% decreased smoking after the first screening. It was more typical in younger participants (<65 years). 64% of the participants thought that early detection of lung cancer improves survival. Healthy lifestyle was common among participants—including exercising, healthy diet and the use of dietary supplements. 39% spent more attention on their diet and the use of dietary supplements increased with 16% after the first screening. According to these results screening may have a positive effect on people’s lifestyle [15].

Screening has also shown positive effects in the British Family Heart Study. The number of smokers decreased after one year of the screening—19% versus 23%; p<.001 [16]. However, there was no significant difference in the ratio of smokers before the screening and three years later in the OXOCHECK Study—25% versus 26%; p=.44 [17,18].

The aim of the study was to determinate the differences between the participation rate of smokers and non-smokers in lung cancer screening among volunteers representing the adult population. What conclusions can be made for more effective communication strategy? Can the rate of smoking be determined, which needs another (way, type, etc.) communication strategy to increase the participation rate in the screening.

## Methods

**Figure 1:**
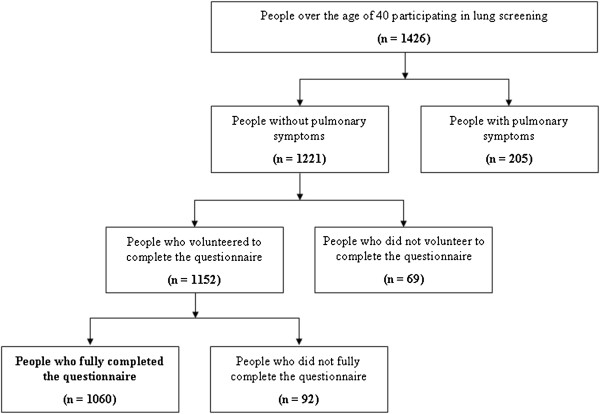
Participants of the survey.

In the given period of time (from May to August 2012) out of 1426 people who participated in the lung screening program 1,060 adult volunteers (331 males/31.2%/ and 729 females/68.8%/, average age 54.0±9.3 years), completed fully and anonymously (74.3%) our questionnaire that contained 28 questions (see Figure 1). We edited the questionnaire on the basis of validated questionnaires of the WHO. It contained open-ended and close-ended questions.

*Inclusion criteria:* (1) voluntary participation in lung screening, (2) completing the questionnaire voluntary and fully, (3) asymptomatic participants (no pulmonary symptoms).

*Exclusion criteria:* (1) not fully completed questionnaire, (2) pulmonary symptoms.

**Figure 2:**
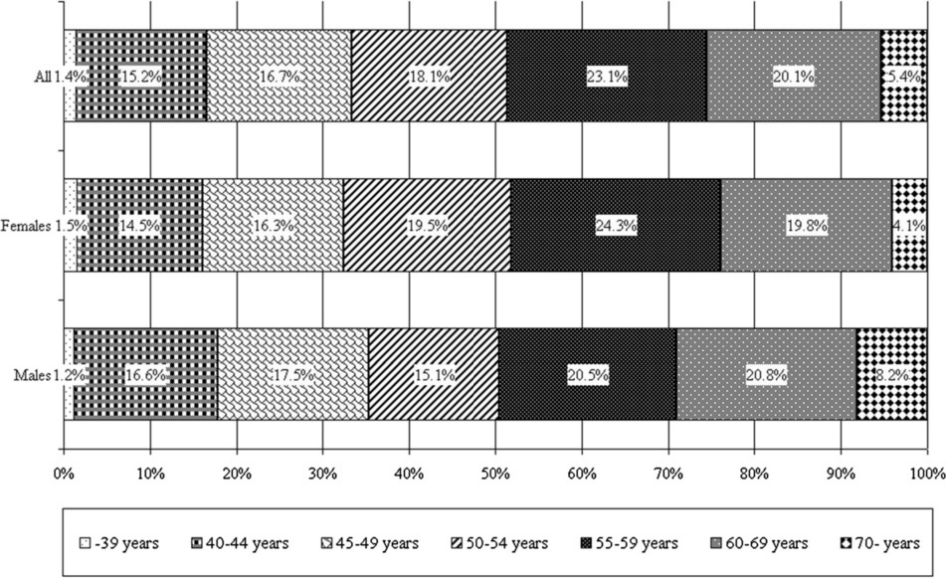
Distribution of participants according to age (n=1,060).

There is no significant difference in the average age of the participant males and females (Table 1) and also the distribution of age (see Figure 2). The survey represents the adult population of Somogy County (n=170,000) according to age and place of residence.

**Table 1:** Characterization of participant according to age

**Age**	**Males**	**Females**	**All**
	**(n=331)**	**(n=729)**	**(n=1,060)**
Mean	54.5 years	53.8 years	54.0 years
Standard deviation	9.8 years	9.1 years	9.3 years
Min.	29.0 years	18.0 years	18.0 years
Max.	83.0 years	95.0 years	95.0 years
Median	54.0 years	54.0 years	54.0 years

Average, standard deviation, minimum, maximum and based on these the ranges of variation were calculated for the each groups. 95% confidence intervals were calculated, we applied two-sample t-tests and univariate and multiple linear regression analysis. Parameters were analyzed not only for the whole population but also in age sub-groups.

Data were analyzed by using SPSS v20.0.0.

Ethical approval was issued by “Kaposvári Egyetem Egészségügyi Centrum Etikai Bizottsága [In Hung.] = Ethical Committee of Kaposvár University Health Center” for this study corresponding to the current national and international ethical laws and guidelines. The reference numbers are: EC 611/2012. Copies of the written documentations are available for review by the Editor-in-Chief of this journal.

## Results

### Lung screening-related attitudes

**Figure 3:**
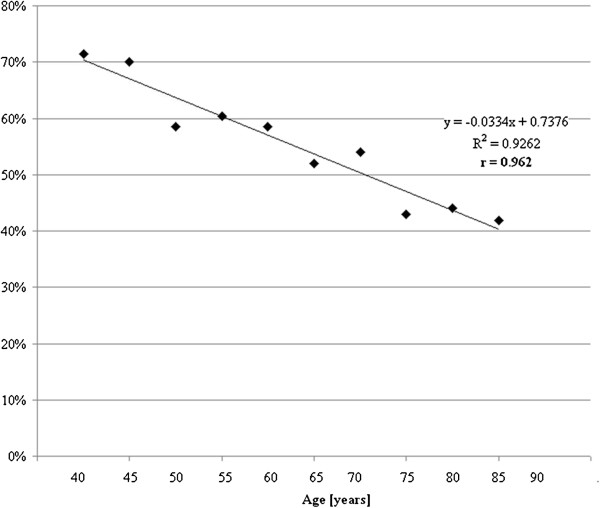
Age and participation in screening.

We analyzed the average screening visits with line up the participants according to age and divided them into ten equal groups. We found that in younger age groups participation in screening is more frequent (Table 2). People less and less believe in it with aging (see Figure 3).

**Table 2:** Annual average participation in lung screening according to deciles

**Decile**	**Average age**	**Average participation in screening**
1	39.13	71.4%
2	43.77	70.0%
3	49.33	58.6%
4	52.16	60.4%
5	54.68	58.6%
6	56.61	52.0%
7	58.55	54.0%
8	61.36	43.0%
9	65.18	44.0%
10	74.47	41.9%

We examined the participants according the level of education and we found that people with the lowest education level participate in screenings the least: only 37.5% participate in screening annually, while from higher educational level groups 60.8% participate annually.

The questionnaire contained questions about the opinion of the respondent about the effectiveness of the screening. Interestingly the respondents who did not exactly know the purpose of the screening programs and they answered with “I do not know” (58.0%) participate in screening rarely, not those who think that it is not effective.

### The ratio of smokers, smoking rate

**Figure 4:**
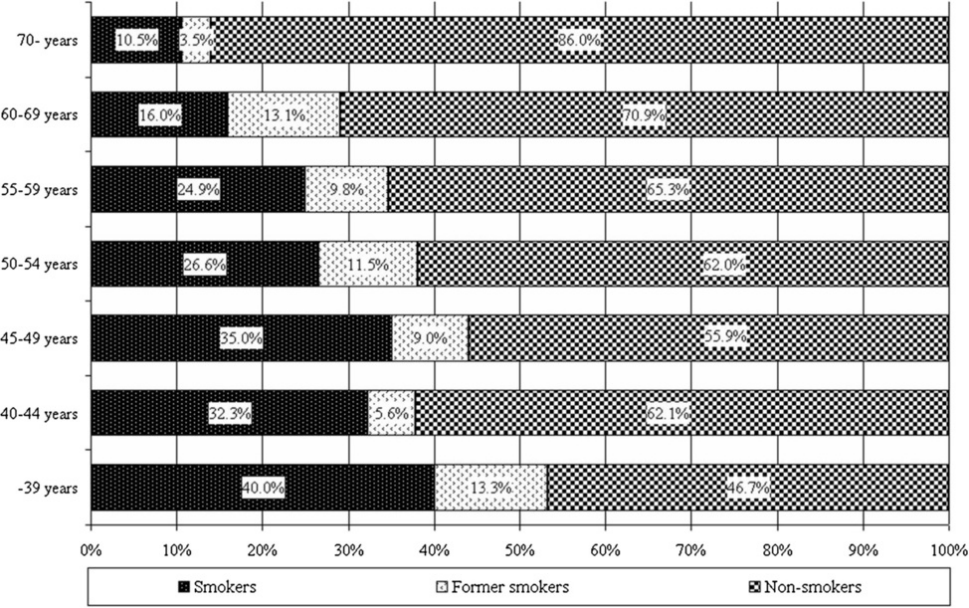
Ratio of smokers in the different age groups (n=1,060).

**Figure 5:**
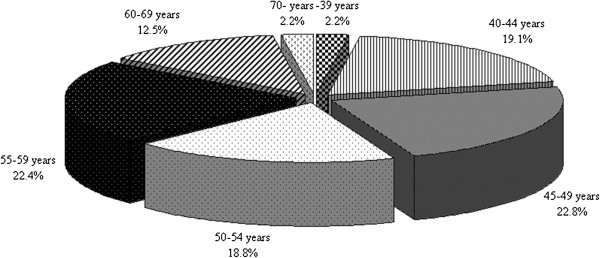
Distribution of smokers according to age (n=272).

25.7% of the respondents identified themselves as smokers (n=272), 47.4% of them started to smoke before the age of 18. All of the smokers smoked a week before the survey. The highest ratios of smokers were in the age groups of 39 year olds or younger and in the group of 45-49 year olds (see Figure 4). The highest proportion of smokers was in the group of 45-49 year olds (see Figure 5).

66.2% of the smokers smoked at least a half pack of cigarette per day, 19.1% smoked at least one pack of cigarette (19 cigarettes) per day. Most people (46.9%) smoked half–one pack of cigarette per day. The average pack-year of cigarette smoking was 28.5±17.8 (it varied between 2.0 and 112.0 pack years) for smokers; it was 34.0±21.6 (it varied between 2.0 and 87.0 pack years) for former smokers.

64.6% of the respondents have never smoked, while 9.7% were former smokers.

### Smokers versus non-smokers

Close relationship was not found between participation in screening and smoking. Although non-smokers participate less frequently in screening but the difference is statistically not significant. However if we analyzed only smokers we found interesting results. Those who smoked less (maximum 10 cigarettes/day) participate more frequently in screenings (72.8%) than heavy smokers (54.4%).

**Figure 6:**
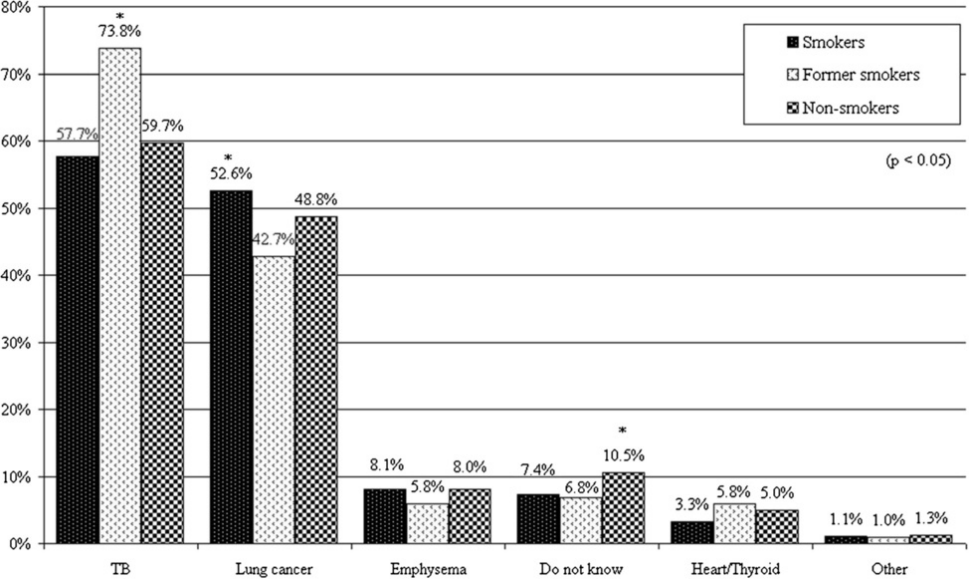
Answers for the question “What do you think, which disease detection is the most important in lung screening?”.

For the question *“What do you think, which disease detection is the most important in lung screening?”* former smokers answered TB (tuberculosis) in significantly (p < .05) higher rate, while smokers answered lung cancer in also significantly (p<.05) higher rate (see Figure 6). The ratio of respondents with the answer “I do not know what disease detection is the most important in lung screening” was the highest among non-smokers. 73.8% of non-smokers thought that the most important is the detection of TB, 52.6% of smokers thought the same about lung cancer, 48.8% of non-smokers thought the same. 42.7% of former smokers considered the detection of lung cancer as the most important in lung screening.

**Figure 7:**
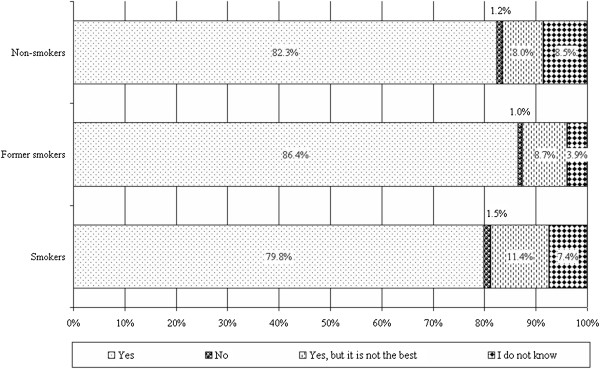
Perceived effectiveness of lung screening in early detection of pulmonary diseases.

Mostly former smokers considered lung screening as an effective method for early detection of pulmonary diseases (86.4%). High proportion of both smokers and non-smokers (7.4% and 8.5%) could not give a substantial answer for this question (see Figure 7).

**Figure 8:**
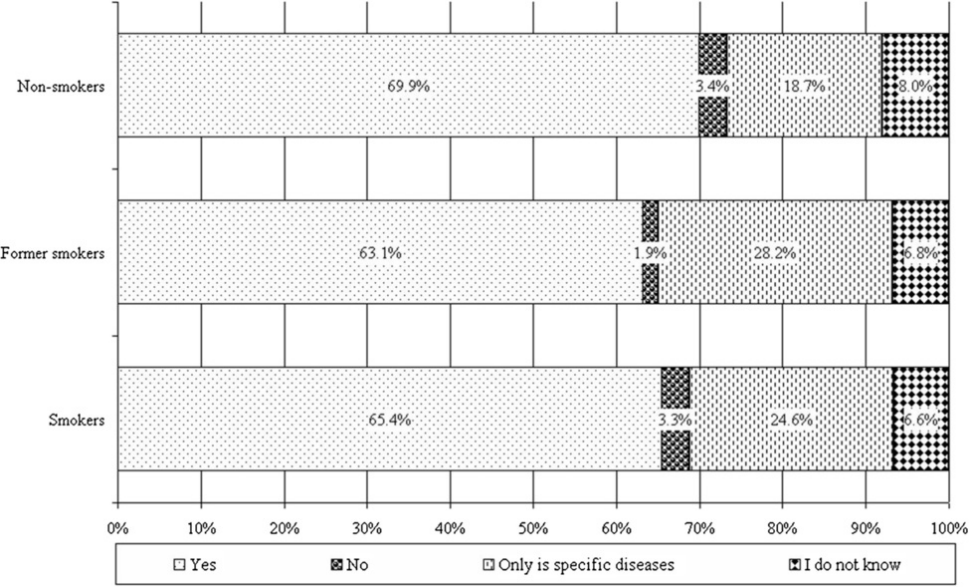
The distribution of answers for the question ”With regular lung screening serious pulmonary diseases can be avoided”.

The highest proportion who thinks that “with regular lung screening serious pulmonary diseases can be avoided” is from non-smokers (see Figure 8).

### Information sources

Those participants who obtain information from more sources significantly more frequently (p < .05) go to screening: maximum from one source 53.9%, two-three sources 59.6%, four or more sources 71.6%.

In general, the most important sources (41.0%) of information are the professionals of the general practitioner’s office (and also the information materials that may find there). Habit also plays an important role in every third respondent (35.5%) to participate in lung screening. Every fifth person (19.9%) uses TV or radio as a source of information. The other screenings played a more important role (5.4%) in the participation of lung screening than internet (3.5%) or school (0.4%).

**Figure 9:**
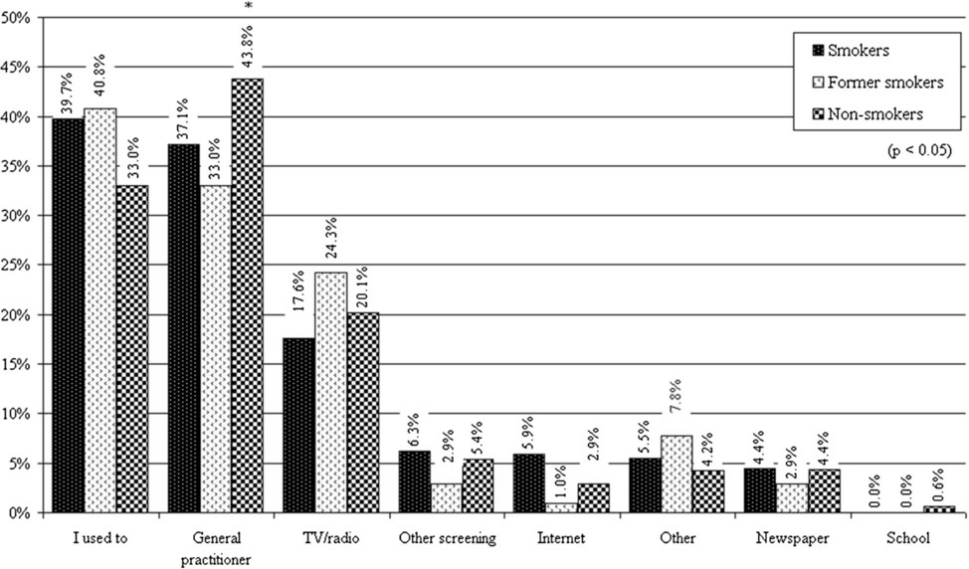
“Where/Who told you that you should go to lung screening?” incidence of the answers of the sub-samples.

There are major differences between the information sources of the three sub-samples (smokers, former smokers, non-smokers), so significantly higher proportion of non-smokers were informed by the general practitioner about the participation in lung screening, while most of the smokers and former smokers participated due to habit (See Figure 9).

**Figure 10:**
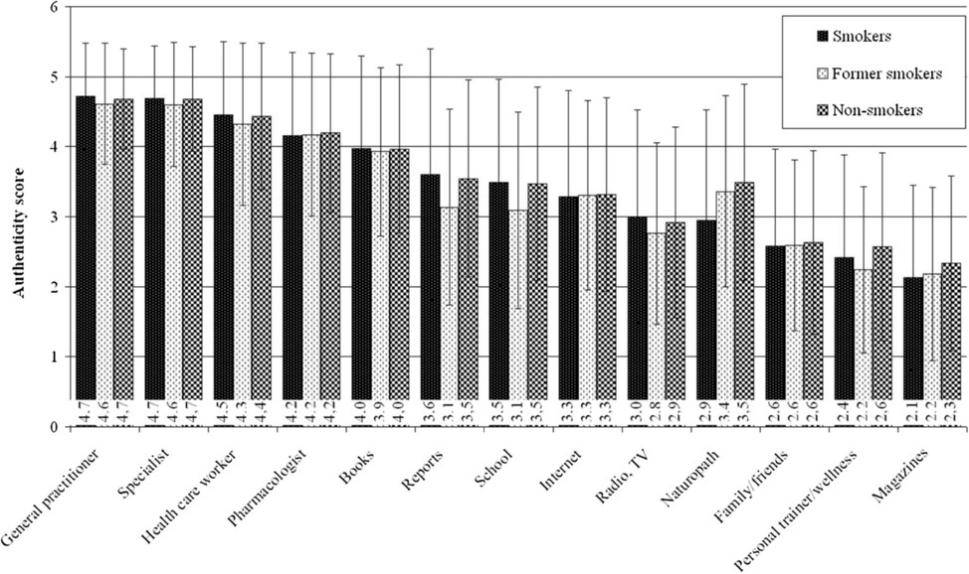
**Accuracy of different sources.***Legend:* 1: least authentic, 5: most authentic.

Smokers mostly considered the general practitioner or the physician (4.7-4.7) and the health professional (4.5) to be accurate. Former smokers and non-smokers answered the same, but in a 1 to 5 rating scale the achieved scores were lower than the scores that smokers gave (see Figure 10). In the accuracy of pharmacists there were no significant differences in the three sub-samples, and also in the accuracy of the other sources.

### Participation in lung screening

**Figure 11:**
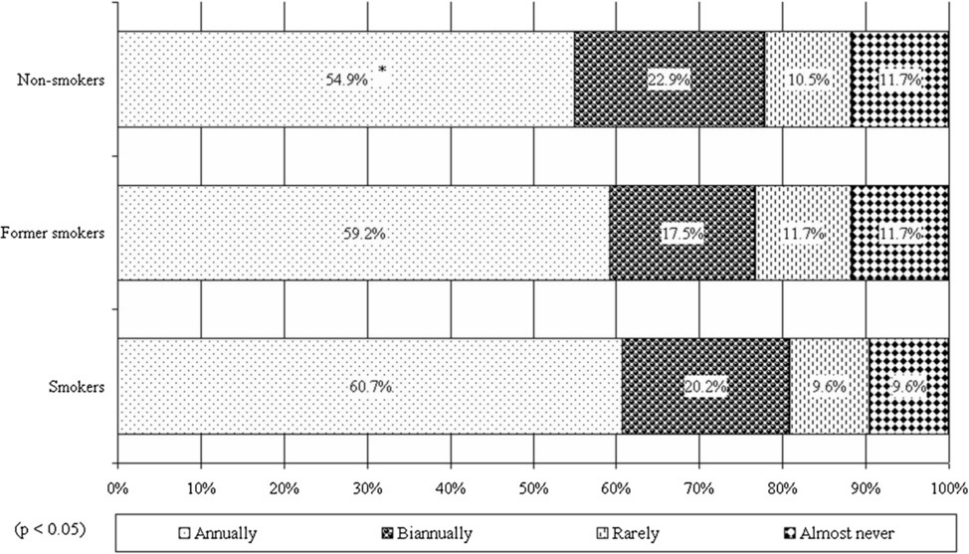
Frequency of participation in lung screening of smokers and non-smokers.

Non-smokers took part in lung screening annually in significantly (p<.05) lower proportion (54.9%), 60.7% of the smokers, 54.9% of non-smokers went annually to the screening, further 17.5%-22.9% went biannually. There is no significant difference between the tendencies of the participation frequency of the two groups. (see Figure 11).

There is a strong correlation (r=.38; p=.01) between the frequency of participation in lung screening and the number of cigarettes smoked per day. Also the participation in other screenings (breast, prostate, osteoporosis, etc.) shows significant correlation (r=.27; p<.05).

With the increase of age the participation rate of non-smokers in lung screening significantly increases per decades. While in the 41-50 and 51-60 years age groups the participation of smokers (less than half pack/day) was typical, in older age groups only smokers who smoke at least half pack per day took part in the screening. 92.1% of smokers and 100% of former smokers read/heard about smoking caused lung cancer. (The ratio in non-smokers was 94.6%).

## Discussion

The participation rate of non-smokers is higher in lung screening than the rate of non-smokers in the population. Similar results were found in the National Lung Screening Trial: according to sex and pack-year history participants represented the population of the United States, but their average age was younger than the population, they were better educated and the ratio of smokers was lower [17]. However, every tenth smoker in the United States is diagnosed with lung cancer [18].

Significantly (p<.05) lower ratio of smokers, particularly men, from the 50-54 years and older age groups participated voluntary in lung screening. In these age groups the currently available information channels and forms of communication are not sufficient.

The knowledge of former smokers about smoking and its harms is wide enough. After they stop smoking their health behavior and health consciousness becomes higher than smokers and non-smokers. We can conclude this from the further results, such as participation in other screenings, BMI calculated from measured parameters, data about nutrition and exercise.

Surprisingly, former smokers referred to school studies in the highest proportion whereas previously they smoked (60% of them became regular smoker before the age of 18).

Smokers significantly more frequently took part in lung screening annually. It is positive—in contrast to the data from the literature [19]—that the participation rate of former smokers is higher than non-smokers, it is just a bit lower than the participation rate of smokers—both in annual and biannual participation. So the “protection” that they might assume to develop after they stop smoking could not be proved according to the results.

There is a strong correlation (r=.33; p=.01) between the number of cigarettes smoked per day and the first time of going to lung screening (“How old were you at the time of your first lung screening?”). This fact and also the similar result about the number of cigarettes smoked per day and the frequency of participation in lung screening shows that a part of smokers takes seriously lung screening, in addition heavy smokers took part more frequently in the screening.

At the same time we have no data about smokers who do not participate, why do they not participate. Furthermore, there is negative correlation between the time (age) of the first cigarette and the annual participation in screening. It is important that our study showed that higher proportion of smokers know that participation is not free for everyone. And higher proportion thought that lung screening is a radiation exposure risk. They also believe that the chest radiography is not an outdated or less effective method in lung screening. The unclear data suggest that smokers need clear, concise messages to know why they should regularly undergo lung screening.

## Conclusions

The aim of this preliminary study was to determinate the differences in the participation rate of smokers and non-smokers in lung cancer screening and to determine the communication strategies to increase the participation rate.

We found that smokers significantly more frequently took part in lung screening annually. It is positive that the participation rate of former smokers is higher than non-smokers, it is just a bit lower than the participation rate of smokers—both in annual and biannual participation. The participation rate of non-smokers is higher in lung screening than the rate of non-smokers in the population.

In order to summarize and evaluate the experiences we can state that besides the high-tech achievements, doctors have a major role in the communication with the targeted groups.

## Competing interests

The authors declare that they have no financial and non-financial competing interests.

## Authors’ contributions

MM produced the imaging analysis, statistical correlations and initial draft manuscript, GB and MR designed and coordinated the clinical interviews with all patients, ZSL provided statistical analysis and interpretation, JS and IR conceived of the study design and coordinated technical assistance. All authors contributed significantly to the interpretation of the data as well as read and approved the final manuscript.

## Pre-publication history

The pre-publication history for this paper can be accessed here:

http://www.biomedcentral.com/1471-2458/13/914/prepub

## References

[B1] Youlden DR Cramb SM Baade PD The international epidemiology of lung cancer: geographical distribution and secular trends J Thorac Oncol 2008 3 819 831 10.1097/JTO.0b013e31818020eb 18670299

[B2] KSH [Pulmonary diseases] Stat Tükör 2009 3 1 3 [In Hung.]

[B3] Jaklitsch MT Jacobson FL Austin JH Field JK Jett JR Keshavjee S MacMahon H Mulshine JL Munden RF Salgia R Strauss GM Swanson SJ Travis WD Sugarbaker DJ The American Association for Thoracic Surgery guidelines for lung cancer screening using low-dose computed tomography scans for lung cancer survivors and other high-risk groups J Thorac Cardiovasc Surg 2012 144 33 38 10.1016/j.jtcvs.2012.05.060 22710039

[B4] Fact Sheet – IARC 2008 Retrieved January 16, 2012 from http://globocan.iarc.fr/ (Select a cancer: Lung)

[B5] WHO Media Center Cancer Fact Sheet. No. 297 2012 Retrieved December 3, 2012 from http://www.who.int/mediacentre/factsheets/fs297/en/

[B6] Didkowska J Manczuk M McNeill A Powles J Zatonski W Lung cancer mortality at ages 35-54 in the European Union: ecological study of evolving tobacco epidemics BMJ 2005 331 189 192 10.1136/bmj.331.7510.189 16037450 PMC1179761

[B7] Dela Cruz CS Tanoue LT Matthay RA Lung Cancer: epidemiology, etiology and prevention Clin Chest Med 2011 32 605 644 10.1016/j.ccm.2011.09.001 22054876 PMC3864624

[B8] Torok S Hegedus B Laszlo V Hoda MA Ghanim B Berger W Klepetko W Dome B Ostoros G Lung cancer in never smokers Future Oncol 2011 7 1195 1211 10.2217/fon.11.100 21992731

[B9] Lung cancer mortality statistics 2012 Retrieved November 7, 2012 from http://www.cancerresearchuk.org/cancer-info/cancerstats/types/lung/mortality/uk-lung-cancer-mortality-statistics

[B10] Bach PB Mirkin JN Oliver TK Azzoli CG Berry DA Brawley OW Byers T Colditz GA Gould MK Jett JR Sabichi AL Smith-Bindman R Wood DE Qaseem A Detterbeck FC Benefits and harms of CT screening for lung cancer: a systematic review JAMA 2012 307 2418 2429 10.1001/jama.2012.5521 22610500 PMC3709596

[B11] McLoud TC Initial results of the National Lung Cancer Screening Trial Cancer Imaging 2011 11 S85 10.1102/1470-7330.2011.9021

[B12] Rampinelli C Preda L Maniglio M Sirica L Travaini LL Veronesi G Bellomi M Extrapulmonary malignancies detected at lung cancer screening Radiology 2011 261 293 299 10.1148/radiol.11102231 21828191

[B13] Pastorino U Rossi M Rosato V Marchianò A Sverzellati N Morosi C Fabbri A Galeone C Negri E Sozzi G Pelosi G La Vecchia C Annual or biennial CT screening versus observation in heavy smokers: 5-year results of the MILD trial Eur J Cancer Prev 2012 21 308 315 10.1097/CEJ.0b013e328351e1b6 22465911

[B14] Brennan P Crispo A Zaridze D Szeszenia-Dabrowska N Rudnai P Lissowska J Fabiánová E Mates D Bencko V Foretova L Janout V Fletcher T Boffetta P High cumulative risk of lung cancer death among smokers and nonsmokers in Central and Eastern Europe Am J Epidemiol 2006 164 1233 1241 10.1093/aje/kwj340 17032696

[B15] Zafar Y Kleykamp B Foraida M Neu J Buncher R Shipley R Howington J Characteristics and motivations of participants in a lung cancer screening study J Clin Oncol 2004 22 1032 Retrived from http://meeting.ascopubs.org/cgi/content/abstract/22/14_suppl/1032

[B16] Wood DA Kinmonth AL Davies GA Yarwood J Thompson SG Pyke SDM Kok Y Cramb R Le Guena C Marteaua TM Durrington PN Randomised controlled trial evaluating cardiovascular screening and intervention in general practice: principal results of British family heart study BMJ 1994 308 313 320 10.1136/bmj.308.6924.313 8124121 PMC2539278

[B17] OXOCHECH Study Group Effectiveness of health checks conducted by nurses in primary care: final results of the OXCHECK study. Imperial Cancer Research Fund OXCHECK Study Group BMJ 1995 310 1099 1104 7742676 PMC2549499

[B18] Deutekom M Vansenne F McCaffery K Essink-Bot M Stronks K Bossuyt PMM The effects of screening on health behaviour: a summary of the results of randomized controlled trials J Public Health 2010 33 71 79 10.1093/pubmed/fdq050 20667898

[B19] Aberle DR Adams AM Berg CD Clapp JD Clingan KL Gareen IF Lynch DA Marcus PM Pinsky PF National Lung Screening Trial Research Team Baseline characteristics of participants in the randomized National Lung Screening Trial J Natl Canc Inst 2010 102 1771 1779 10.1093/jnci/djq434 PMC2994863 21119104

